# The Body as Evidence for the Nature of Language

**DOI:** 10.3389/fpsyg.2018.01782

**Published:** 2018-10-29

**Authors:** Wendy Sandler

**Affiliations:** Sign Language Research Laboratory, University of Haifa, Haifa, Israel

**Keywords:** sign language, compositionality, embodiment, language emergence, language evolution, emotion

## Abstract

Taking its cue from sign languages, this paper proposes that the recruitment and composition of body actions provide evidence for key properties of language and its emergence. Adopting the view that compositionality is the fundamental organizing property of language, we show first that actions of the hands, face, head, and torso in sign languages directly reflect linguistic components, and illuminate certain aspects of compositional organization among them that are relevant for all languages, signed and spoken. Studies of emerging sign languages strengthen the approach by showing that the gradual recruitment of bodily articulators for linguistic functions directly maps the way in which a new language increases in complexity and efficiency over time. While compositional communication is almost exclusively restricted to humans, it is not restricted to language. In the spontaneous, intense emotional displays of athletes, different emotional states are correlated with actions of particular face and body features and feature groupings. These findings indicate a much more ancient communicative compositional capacity, and support a paradigm that includes visible body actions in the quest for core linguistic properties and their origins.

## Introduction

Sign languages and spoken languages differ dramatically in the physical modality of transmission. Despite this difference, since sign languages have been taken seriously as full natural languages, investigators have placed the emphasis on the numerous similarities between the two systems. In *Sign Language and Linguistic Universals* (Sandler and Lillo-Martin, 2006), all chapters but one adopt theories devised on the basis of spoken language to analyze the morphology, phonology, prosody, and syntax of sign languages. Though the physical manifestations of linguistic properties are duly described, the research paradigm works from linguistic theory to its manifestation by the body – from the linguistic mechanisms in the mind out to the body. Only the final chapter of the book deals with so-called modality effects that distinguish the form of sign language from that of spoken language. Here the direction of investigation is reversed. Working from the body to language, from the outside in, I bring together a range of diverse studies to show that the recruitment and composition of body actions provide direct evidence for linguistic properties and their emergence.

Since the beginning of linguistics, the main object of study has been the structure and arrangement of words. This focus has been attributed to the technology of writing, which made it possible to record these parts of language, so that they could be studied scientifically (Downing et al., 1992). As a result, the elements that can be recorded in writing, the principles behind them, and the meanings associated with them, became the primary data. Among the effects of writing systems is the segmental view of the language signal as beads on a string (see e.g., De Saussure, 1959; Aronoff, 1992). The technology of writing systems facilitated linguistic analysis, and linguists have made much progress over the last century and more using words and their arrangement as data.

Writing has undoubtedly advanced civilization, but it is not a component of the language faculty. According to *Ethnologue* (Simons and Fenning, 2018), fewer than half of the world’s languages have writing systems, and for most of those that do, there are large populations of speakers who are illiterate in the language, and have not achieved what Gough and Hillinger (1980) famously called ‘an unnatural act’ – learning to read. Furthermore, standard written languages like Chinese, English, or Hindi, almost never represent a person’s actual spoken language. The human language capacity is independent of the written word. The fact that it is possible to convey much of the (spoken) language message in writing is of interest, but it is also deceptive.^1^

The language faculty is intimately entrenched in the body – not only in the voice, but in the face, the hands, and the torso as well. In recent decades, technology for recording language has advanced greatly, and it is now easy to capture and study both the auditory and visual signals that are the physical substance of language – what we actually produce and perceive. These advances have influenced the study of phonetics, phonology, and intonation, fostering new approaches such as articulatory phonology (Browman and Goldstein, 1992). Through video technology, we can now observe gestures and facial expressions, facilitating the much younger but thriving field of co-speech gesture (McNeill, 1992; Kendon, 2004; Müller et al., 2013, 2014; Church et al., 2017). These technological advances allow us to study the interaction between the auditory and visual domains in spoken language. By including visually perceived bodily signals in our understanding of human language, we put language back in the body, and humans in their ecological evolutionary setting.^2^

In the natural and spontaneous languages of deaf communities, there is no language at all without the visible bodily signal. Technological advances have also made it possible to study these languages rigorously; for example, the early and seminal research of Klima and Bellugi (1979) relied partly on videotaped data. Sign languages emerge spontaneously and relatively quickly whenever deaf people have an opportunity to communicate regularly (e.g., Senghas, 2003; Sandler et al., 2005), and even individual deaf children in hearing, speaking households create gestural systems with the seeds of linguistic structure (Goldin-Meadow, 2003). It is now accepted that sign languages are a manifestation of a universal human linguistic endowment. It follows that they should not be regarded as extraneous or peripheral, but rather as fundamental to our understanding of language.

Taking its cue from sign languages, this article pulls together results from a range of studies to support the proposal that the recruitment and composition of body actions count as primary evidence for linguistic properties and their emergence. This approach has two aims. The first relates to sign language and co-speech gesture; and the second relates to all language. The first aim is to motivate a model of the relation between linguistic functions and bodily actions in sign languages, and a principled way of relating that model to co-speech gesture. The second is to give the human body a focal role in the pursuit of knowledge about core properties of language, how they interact, how they emerge in new languages, and how they evolved. The approach complements and supplements those that study only mind-internal computational manipulations that create language structure (see e.g., Chomsky, 2007).^3^

A single thread that unifies all modern linguistic research is that the human language capacity is rooted in our ability to communicate compositionally. Compositionality was first introduced by Frege (1914/1979) as a constraint on the relation between syntax and semantics (see Hinzen et al., 2012). This versatile capacity is a robust human trait. Other species, such as non-human primates, can certainly command compositionally organized cognitive operations and social systems, which may indeed have provided primordial underpinning for compositional expression (see the section on Language Evolution below). However, to date, evidence for compositionality in the communicative capacity of other species is scant.^4^ The version of the compositionality principle assumed here is given in (1).

(1) The compositionality principle (Szabó, 2012, p. 71).
  The meaning of a complex expression is determined by the meanings its constituents have individually and the way those constituents are combined.

Complex words can be understood in terms of their component parts, and the same is true of phrases, clauses, complex sentences, and so forth. It is understood that each component can be recombined with other components, within the constraints of the system, to create new complex forms. Though compositionality does not exhaustively account for all of language structure, the basic principle is robust and results in productivity and creativity in the language of humans, and of humans alone.

In what follows, motivation for the body-as-evidence approach, in which the body and compositionality figure prominently, comes from four directions: (1) established sign languages, (2) language emergence (of which the only empirical data are from sign languages), (3) gesture, and (4) communicative displays of intense emotion in a human compositional system that is far more ancient than language.

The idea that sign languages are fully fledged linguistic systems at all levels of structure is by now widely accepted across the scientific community (Stokoe, 1960; Klima and Bellugi, 1979; Sandler and Lillo-Martin, 2006; Pfau et al., 2012). But inadvertently, indirectly, and somewhat myopically, the written word and the language-as-computation paradigm have dominated sign language research, as they have that of spoken language.

Sign languages, by their very nature, convey linguistic information directly through articulations of different parts of the body – an advantage for linguistic analysis that is typically overlooked. It can be no accident that (apart from differences in detail of the kind that any grammatical system would exhibit, due to conventionalization and automaticity) unrelated sign languages tend to achieve this kind of structuring in very similar ways. The section on Established Sign Languages demonstrates that what I call the Grammar of the Body, which reflects universal elements of meaning and structure in a way that speech cannot. The role of iconicity in this system, all the way down to and including the phonology, is addressed, and considered in light of recent demonstrations of iconicity in spoken language.

Another advantage offered by sign languages is their youth. It is only in sign languages that language emergence, the topic of the section on The Composition of Language Emergence can be observed empirically, since it is only these languages that can emerge de novo at any time. In the initial stages of language emergence, we do not see the sophisticated associations between body and language form found in established sign languages. Research on Al-Sayyid Bedouin Sign Language (ABSL), an emerging language in a Bedouin village, summarized in its own section below, suggests that the gradual recruitment of parts of the body, as well as the refinement of these articulations and their interactions over time, reveals the way in which linguistic organization emerges, step by step (Sandler, 2012a). The body-based approach advocated here reveals which components of language organization arise earlier than others. We infer that these early components are critical for successful linguistic interaction. The next section then summarizes support for this perspective from another young sign language that arose under different social and linguistic conditions, Israeli Sign Language. Broadly speaking, sign languages tend to have similar body-to-language representations, suggesting that they derive from a universal, gestural base common to all of us.

The section, Gesture briefly cites related observations from the field of co-speech gesture studies. The goal is to show how the Grammar of the Body found in sign languages is tapped by gesture as well, supporting the view that gesture provides a universal base for the systematic and constrained system underlying sign languages.

Since compositional communication is very limited or non-existent in other species (see footnote 4), but robust in humans, the question of its evolutionary origins is of interest. Some comments about different views of language evolution open the section that probes The Roots of Compositional Expression in Intense Emotional Displays. A search for the foundations of bodily compositionality leads to the study of body signals in humans that are communicative but non-linguistic, and that have internal compositional organization: body displays of intense emotion. We review our recent experiments, which analyze displays of winning and losing athletes (Cavicchio et al., 2018). Interpretation of these displays – minutely coded for features of face and body – form the basis of a compositional model of the expression of emotion, illustrated for the first time here by idealized computer-generated 3-D images. This evidence from the body suggests ancient roots for compositional communication in humans.

The final section brings together these strands of research, to offer a basis for incorporating the body into future investigations of the nature of language.

## Body and Language Structure in Established Sign Languages

One of the most important differences between signed and spoken language is that, in sign language alone, movements of articulators (of the face, hands, and body) correspond directly to specific linguistic functions. This situation is quite unlike speech, in which movements of the vocal apparatus in themselves typically do not signify linguistic categories directly. That is, the relation between linguistic form and movement of any part of the vocal tract and the resulting acoustic signal is indirect. Across sign languages, despite expected grammatical differences, the same fundamental correspondences between bodily actions and types of linguistic functions seem to hold. This strongly suggests that sign languages are tapping deeper body-meaning correspondences, common to us all, and converting them into rule-governed linguistic systems.

The correspondences are identifiable and reveal compositional structure inherent in signed words themselves as well as in the organization of sign languages at higher levels. The following sections look selectively at the linguistic roles played by the hands, the face, the torso, and the non-dominant hand independently. The evidence points to a deeper source: the relation between communicative conceptualization and the body – for all of us.

The rich cross-linguistic literature on spoken languages is unfortunately not paralleled in the relatively young field of sign language research. In the discussion that follows, data and analyses are presented from several, often unrelated sign languages. Unless otherwise stipulated, the general characteristics described below are, to the best of my knowledge, representative of sign languages in general. Broader cross-linguistic confirmation and grammatical detail await future empirical research.

### The Hands: Iconicity and Dual Duality of Patterning

**FIGURE 1:**
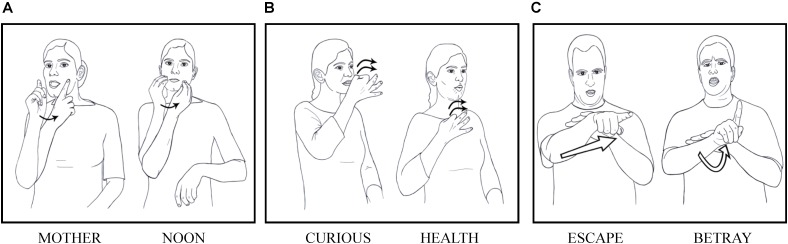
Minimal pairs in Israeli Sign Language. Form left to right: **(Ai)** MOTHER, **(Aii)** NOON distinguished by handshape; **(Bi)** CURIOUS, **(Bii)** HEALTH distinguished by location; and **(Ci)** ESCAPE, **(Cii)** BETRAY distinguished by movement.

In all sign languages, the hand or the two hands together produce forms equivalent to words. Contrary to popular belief that preceded scientific sign language research (e.g., Bloomfield, 1933), Stokoe (1960) demonstrated conclusively that signs are not holistic gestures. They are composed of units of handshape, location, and movement, which make contrasts and in other ways function like the meaningless phonemes and features of spoken language. For example, Figure 1 shows minimal pairs in Israeli Sign Language, distinguished only by features of handshape (1a), location (1b), and movement (1c).

This means that sign languages share with spoken languages the design feature named “duality of patterning” by Hockett (1960); called ‘double articulation’ by Martinet (1960): words in both modalities are comprised of both meaningless (phonological) and meaningful levels of structure. Stokoe’s non-trivial claim has been further investigated, corroborated, and refined by other researchers (e.g., Liddell and Johnson, 1986; Sandler, 1989, 2012b, 2017; van der Hulst, 1993; Brentari, 1998). The handshape, location, and movement units behave like meaningless phonological elements in the sense that their combination is constrained by their form, and they are permuted by typical phonological processes such as assimilation and deletion, which are also oblivious to meaning, targeting and influencing articulatory properties of the elements.

Evidence for a meaningless level of structure is seen in American Sign Language lexicalized compounds, which undergo the standard phonological processes of reduction and assimilation (Liddell and Johnson, 1986; Sandler, 1987, 1989, 2017). The reduction involves deletion of locations and regressive assimilation that affects the shape and orientation of the hand. The resulting compound assumes the optimal form of the prosodic word in ASL: the monosyllable (Sandler, 1999a). What is important here is that the reduction and assimilation processes affect sublexical components because of their form, irrespective of meaning, and in fact often obscure the meaning of the individual members of the compound.

However, in their enthusiasm to demonstrate that sign languages are full languages like spoken languages, researchers often miss generalizations that result from the iconicity that is still present in the formational units of signs. That is, even as the composition and behavior of formational elements in the system tap their form regardless of meaning, the elements themselves can still bear meaning.

Iconicity goes beyond the general impression of the whole sign. A growing body of work has been describing iconic aspects of the sublexical structure of signed words (e.g., Johnston and Schembri, 1999; Fernald and Napoli, 2000; Taub, 2001; Meir, 2002, 2010; Perniss et al., 2010; Padden et al., 2013). We can say that duality of patterning in sign languages is itself double-sided: the elements that are analogous to the meaningless ‘phonemic’ units of spoken language are also often meaningful. Here sign languages and spoken languages depart, because of the iconic opportunities that the manual-visual medium so richly supports.

The semantic composition of words in any language is quite complex, even when the form is morphologically simple (Wunderlich, 2012). For example, Jackendoff (1990) analyzes the concept ‘drink’ as shown in example (2).

(2) Lexical conceptual structure of the word *drink* (Jackendoff, 1990)
  drink: [event CAUSE ([thing]i, [event GO (thing LIQUID]j,
  [path TO ([place IN ([thing MOUTH OF ([thing]i))])])])]

**FIGURE 2:**
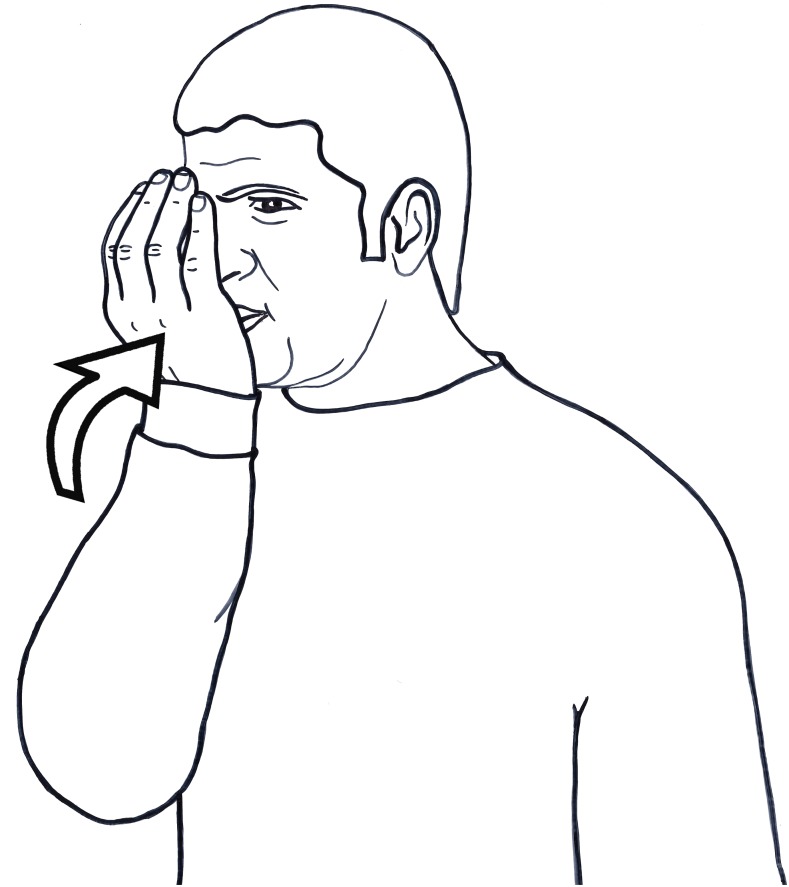
DRINK in Al-Sayyid Bedouin Sign Language (ABSL).

This internal structure is rarely observable in the form of the spoken word itself, e.g., *drink* in English, [⎰ote] in Hebrew, *boit* in French. In any sign language, elements of the internal structure are often reflected directly, and, together, make up the meaning of a sign. Consider the sign DRINK (water) in the emerging sign language of Al-Sayyid, in Figure 2.

The curved hand is a container; the motion reflects causing a substance to go into something; and the mouth as place of articulation is the ‘something,’ the destination. The fact that the ‘something’ is liquid is reflected in the shape of the hand and its orientation with respect to the location, the mouth. Such signs are not pantomimes, but conventionalized signs and specific to ABSL. In ISL, for example, the handshape for DRINK is different from that of ABSL, derived from holding a vessel, while the movement and location still directly reflect the event of moving something liquid into the mouth location.

The sublexical components of handshape, location, and movement, which, as we see here, often retain meaning, combine according to morpho-phonological constraints (rules). These components are not morphemes in the traditional sense, since they do not serve as roots, stems, inflections or derivational elements (but see Lepic, 2015 for a different view). Nevertheless, the components are often motivated, revealing internal semantic structure, and so may be thought of as meaningful phonological elements (see Taub, 2001 for a model of meaningful sign components in ASL). Section “Iconicity in Two-Handed Signs” gives an example of iconicity of phonological elements in two-handed signs, and “Iconicity in Location and Movement” considers the phenomenon in light of recent work on iconicity in spoken language.

#### Iconicity in Two-Handed Signs

Recent comparative work on two-handed signs illustrates the direct relation between the internal semantic structure of a word and its bodily representation. About half of the signs in any sign language are produced with one hand; the other half are two-handed. Previous work on the phonological structure of two-handed signs from different theoretical perspectives have often ignored or downplayed meaning (Battison, 1978; Sandler, 1989, 1993; Van der Hulst, 1996; Crasborn, 2011).

But the selection of two hands rather than one, and of the type of two-handed sign, is often motivated. Comparing lexicons of three unrelated sign languages, we have shown that signs denoting meanings that are essentially plural tend to be two-handed, more than twice as often as chance would predict (Lepic et al., 2016). Specifically, plurality, expressed in relations of composition, interaction, dimension, and relative location among entities or parts of entities, tend to be two-handed in American, Swedish, and Israeli sign languages. A subset of these signs was elicited in Al. Sayyid Bedouin Sign Language, and the results were compatible with findings for the other three sign languages. In these signs, each hand and the interaction between the two represents a component, directly revealing the composition of the concept.

**FIGURE 3:**
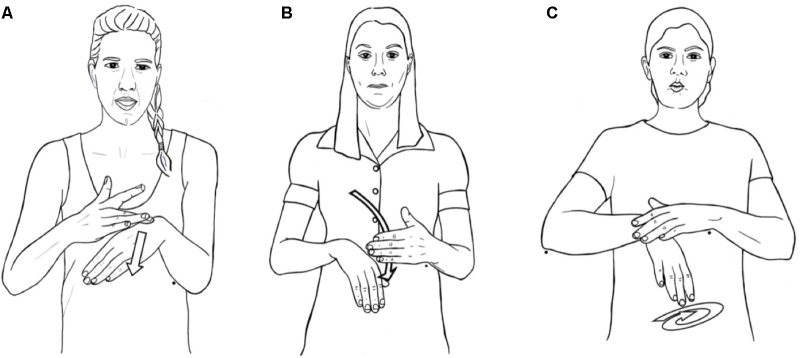
EMPTY in (left to right) **(A)** American, **(B)** Swedish, and **(C)** Israeli sign languages.

For example, the sign EMPTY (Figure 3) in American, Swedish, and Israeli sign languages is unbalanced, that is, non-symmetrical, in all cases. The non-dominant hand represents a surface or container, and the dominant hand signifies its empty or unencumbered state by the type of motion it articulates in relation to the container. The two elements – an object and its empty state – are not equal in EMPTY; it is the empty state that is the salient meaning component in the concept and not the object itself. Only the dominant hand moves to signify emptiness with respect to the non-dominant hand, which signifies the surface or container, and the sign is two-handed and unbalanced in three unrelated sign languages. Enfield (2004) documented similar though unsystematic and gestural use of the two hands in the description of fish traps in Lao.

Here is the crux: particular elements of the cross-linguistic, compositional meanings of concepts, usually not overtly present in the form of spoken words, are often directly revealed in sign languages in similar ways – by the body.

#### Iconicity in Location and Movement

**FIGURE 4:**
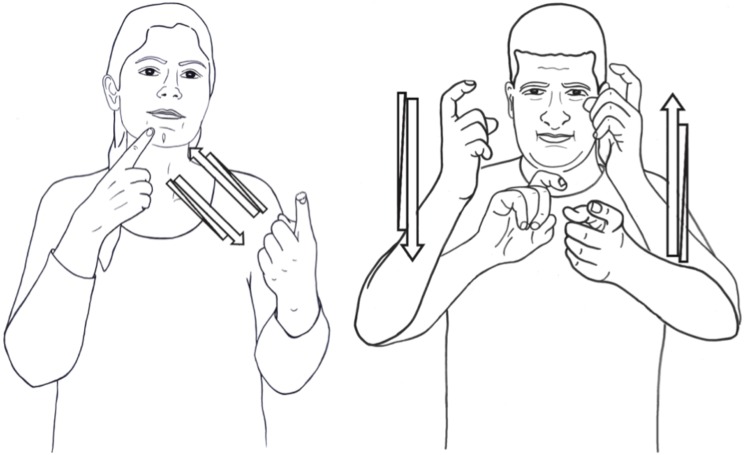
Iconic movement in signs meaning NEGOTIATE in ISL and ABSL.

In most of the examples above, the location category of a sign can also be motivated (Fernald and Napoli, 2000; van der Kooij, 2002). For example, thought processes are typically signed on or near the upper part of the head. Movement patterns are motivated in many signs as well. Figure 4 reveals iconicity in the movement patterns produced by the hand/s: the reciprocal, ongoing activity of negotiating motivates repeated, alternating movement of the two hands. Wilbur (2008), proposes that event structure is directly revealed in the movement pattern of verbs across sign languages. Strickland et al. (2015) provide experimental perceptual evidence from signers across sign languages and non-signers regarding movement and telicity. They write that their results “are highly suggestive that signers and non-signers share universally accessible notions of telicity as well as universally accessible “mapping biases” between telicity and visual form. (2015, p. 1).

A caveat: Not all signs are transparently iconic: many signs are arbitrary in form, and even those with iconic elements are not usually transparent – their meaning cannot be guessed by naïve observers (Klima and Bellugi, 1979). As a language matures, iconicity may diminish and signs may become more arbitrary with respect to their meaning and more constrained in form (Frishberg, 1975 for ASL; Meir and Sandler, 2008 for ISL). Furthermore, not every aspect of lexical conceptual structure is expressed iconically. For example, in the case of DRINK (example 2 and Figure 2 above), the liquid property of what is ingested is only pragmatically inferable.^5^ Different sign languages do not always select the same meaning components for iconic representation. In addition to semantic composition, culture plays a role. If all sign languages selected the same meaning components to represent, there would be only one sign lexicon, rather than hundreds.

Yet meaning is pervasive even in formational units that behave like meaningless phonological elements; it accounts robustly for productive aspects of vocabulary formation and for similarities across sign languages. Words of sign languages, to a much greater extent than those of spoken languages, exhibit what we might call ‘dual duality of patterning,’ and their study across sign languages will have much to reveal about the semantic composition of lexical concepts in human language generally.

#### Iconicity in Spoken Language

Contrary to traditional beliefs about the arbitrary relation between form and meaning in spoken language (De Saussure, 1959), instances of lexical and sublexical form have been found to have an iconic relationship (Blevins, 2012; Dingemanse et al., 2015). Blasi et al. (2016) show that some non-arbitrary associations between form and meaning are even shared across linguistic lineages, suggesting that they are not spread through language contact, but are more basic, and might even have provided an evolutionary base for language tens of millennia ago.^6^ However, the amount of iconicity in sign languages is far greater than in contemporary spoken languages, for two reasons: (1) sign languages, expressed with two visible, anatomically identical articulators, so readily avail themselves of the complex iconic representation necessary for a large vocabulary, and (2) sign languages are very young compared to spoken languages – none of them traceable farther back than 300 years (Kyle et al., 1988). Presumably, a large pool of arbitrary signal-form relations requires time to develop. ^7^

The recent investigations into iconicity in spoken language in fact only serve to reinforce the claim that iconicity in sign languages can reveal universal properties of language that are not – or are no longer – as prevalent in spoken languages. Sign languages teach us that meaninglessness in duality of patterning of human language lies on a continuum and is not absolute.

### The Face

In sign language after sign language, particular aspects of information structure are signaled by the upper face – brows and eyes – and by head position on the front/back axis.^8^ Across sign languages, raised brows and often head forward accompany yes-no (polar) questions, while furrowed brows accompany wh- (content) questions (ASL, Liddell, 1980; Sign language of the Netherlands, Coerts, 1992; British SL, Sutton-Spence and Woll, 1999; other sign languages, Zeshan, 2004). Squinted eyes reliably accompany shared (but not highly accessible) information, another information structuring device in ISL (Dachkovsky and Sandler, 2009; Sandler et al., accepted), and have been observed for the same function in ASL (Dachkovsky et al., 2013) and Danish Sign Language (Engberg-Pedersen, 1993), three unrelated languages.

**FIGURE 5:**
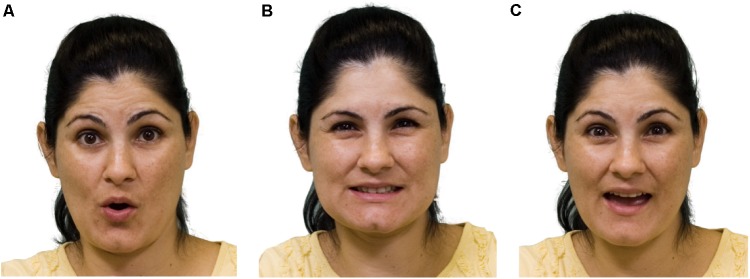
Compositionality of facial intonation in Israeli Sign Language. **(A)** Raised brows for yes/no questions and continuation/dependency. **(B)** Squint for shared but not easily accessible information. **(C)** Raised brows and squint together for a yes/no question about shared information (following Nespor and Sandler, 1999).

In our work, we confirm on functional and distributional grounds the earlier suggestion that facial expressions comprise the intonational component of prosody in sign languages (Reilly et al., 1990), and demonstrate that these signals are compositionally organized (Nespor and Sandler, 1999; Dachkovsky and Sandler, 2009; Sandler, 2010). In spoken language, the vocal cords convey both words and intonation, and different intonational patterns are manifested by fluctuations in frequency of vibration of the vocal cords, sequentially conveyed. This makes it challenging to demonstrate compositionality of intonation in spoken language, though it has been claimed to exist (e.g., Hayes and Lahiri, 1991). In sign languages, intonational signals are conveyed by articulators (such as different parts of the brows and the upper and lower eyelids) that are independent of each other and of the hands, used for words. This means that compositional structure of intonation is clearly revealed by the ways these components simultaneously combine (see Figure 5 below).

While some of the linguistic facial expressions of sign languages are similar to expressions that can also accompany speech (Scherer and Ellgring, 2007; Kidwell, 2013), there is an important difference. In sign languages, these signals are more systematic, both in form and in distribution, and there are some differences across sign languages (Zeshan, 2004; Dachkovsky et al., 2013). Our study of ISL and ASL showed that over 90% of the relevant constituents are characterized by particular linguistic facial expressions and head positions (Dachkovsky and Sandler, 2009; Dachkovsky et al., 2013; Sandler et al., accepted).^9^

The intonational system in sign languages is itself compositional. In Figure 5A below, we see the raised brows of a typical yes-no question, in Figure 5B the squint of shared information, and in Figure 5C, the two intonational units combined, to characterize a yes-no question about shared information, as in *Did you see that movie we talked about last week*?

**FIGURE 6:**
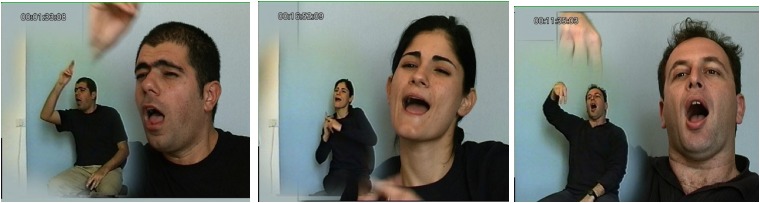
Conventionalized non-intonational ISL mouth adverbial: “for a long time” (from Sandler, 2009).

The lower face is also important in sign languages, but its role is different from that of the upper face. It conveys modification of predicates, meanings such as a ‘for a long time,’ ‘carelessly,’ ‘effortlessly’ (e.g., Liddell, 1980 for ASL, Meir and Sandler, 2008 for ISL and ASL; Sutton-Spence and Woll, 1999 for British SL). It is common that such meanings are conveyed by articulations of the lower face across sign languages that have been studied for this characteristic, although the specific lower face configuration can differ across sign languages (see Meir and Sandler, 2008 for a comparison of lower face modifiers in ASL and ISL). Figure 6 below demonstrates a mouth shape meaning ‘for a long time’ in ISL, taken from retellings by three signers of the same part of a Tweety Bird cartoon, in which the cat and bird fall through the air from a high place (from Sandler, 2009).^10^

### The Head

The whole head also helps to organize information structure, for example, by assuming a particular position (such as forward for questions), or by clearly changing its position to signal a prosodic boundary (Nespor and Sandler, 1999). In the latter case, the head helps to signal dependency between clauses or information units such as topic and comment (Liddell, 1980; Dachkovsky and Sandler, 2009; Sandler et al., 2011). The full sentence example in Figure 8 below shows the head position on either side of the prosodic boundary, in this case, separating the topic from the comment in ‘The little dog that I found last week – ran away.’

### The Torso

**FIGURE 7:**
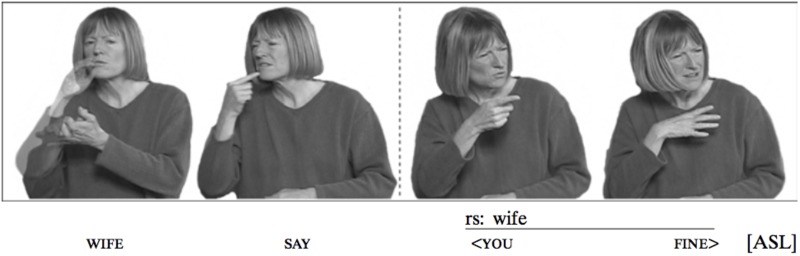
Referential shift of upper body in American Sign Language (reprinted with permission from Lillo-Martin, 2012).

Torso displacement takes different forms, among them shift and tilt. ^11^ A shift in the direction toward which the torso is facing tracks reference and coreference in a discourse. Shift indicates a change in speaker (signer) perspective, sometimes called role shift, and is typically used for direct or indirect quotes in discourse or for what is called constructed action (Lillo-Martin, 1995, 2012; Janzen, 2004; Cormier et al., 2013). Taken together, we can say that torso shift involves assuming the perspective of a character for a stretch of discourse (Quer, 2011; Hermann and Steinbach, 2012; Lillo-Martin, 2012; Schlenker, 2017a,b). In its most overt and full form, this displacement or shift usually consists of positioning the torso so that the chest is facing in a different direction for each perspective, shown in Figure 7.

A tilt, in which the body faces forward but tilts at the waist to one side or the other, can indicate contrastive focus in Sign Language of the Netherlands (Crasborn and van der Kooij, 2013), and can separate constituents in a sentence, most commonly, topic and comment (Dachkovsky et al., 2013 for unrelated ISL and ASL). Torso tilt contrasting the topic from the comment in two intonational phrases is illustrated in Figure 7.

In general, torso movement conveys a contrast of character perspectives or of topics in the common ground. This characterization is broad, and the cross-sign language generalizations we might glean from it must still be confirmed.^12^

### The Non-dominant Hand in Discourse

**FIGURE 8:**
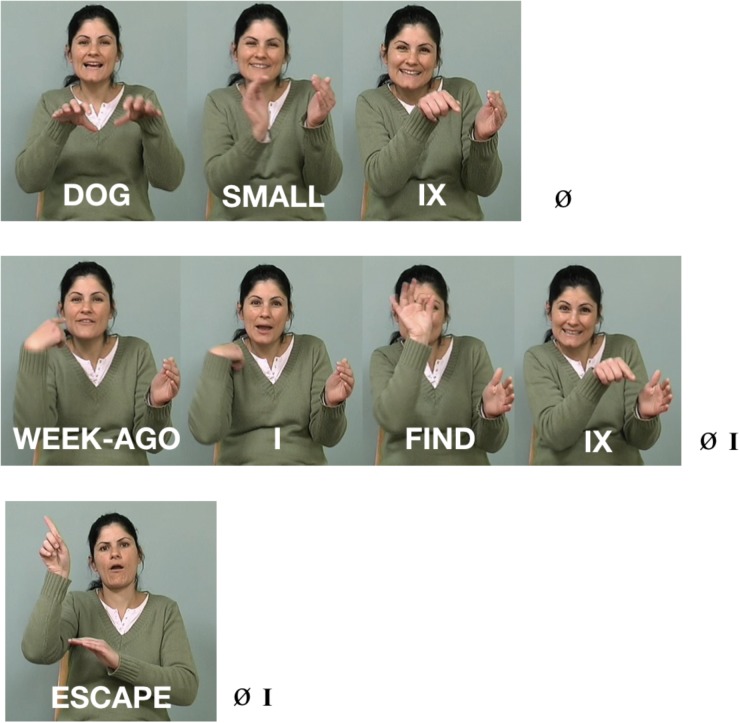
A complex sentence in ISL, “The little dog that I found last week ran away.”

Like other sublexical formational elements, the non-dominant hand can be interpreted as meaning bearing, as seen in Section The Hands: Iconicity and Dual Duality of Patterning above.^13^ As such, it can represent a free classifier (Aronoff et al., 2003; Emmorey, 2003), or it can be dissociated from its two-handed sign, maintaining its shape and position in the signing space, and its inherent meaning, while the dominant hand goes on to produce other signs.^14^ An example of the latter is seen in Figure 8, where the non-dominant hand represents the small dog. In this way, the non-dominant hand marks different kinds of topic continuity, disappearing from the signing space when the discourse topic changes.^15^

### Putting the Body Back Together

If we consider the actions of the body in sign language, and work from body to linguistic structure, general properties of language stand out in high relief. The articulators each mark different linguistic functions, and they are physically independent of one another, which is also an advantage for analysis. This independence makes it possible to incorporate a good deal of simultaneity of structure in sign language utterances, where spoken languages are much more confined to linearity. The relation between articulations and functions in sign languages is not exhaustively 1:1; the same articulation can manifest more than one linguistic function. However, when communication is exclusively visual, and is conveyed by a large number of articulators whose movements are directly perceivable and often simultaneous, the result is a system that can be both complex and transparent at the same time. This transparency often reveals general linguistic properties that are opaque or covert in spoken languages. In sign languages, complex linguistic composition can be seen at a glance.

Putting it all together, Figure 8 above shows a sentence that means, ‘The little dog that I found last week ran away.’ Here is the gloss, in which ‘IX’ stands for ‘index,’ typically a pointing pronominal sign^16^; subscript ‘I’ stands for an intonational phrase; and subscript Ø stands for a more minor, phonological (or intermediate) phrase: [[DOG SMALL IX] _Ø_ WEEK-AGO I FIND IX] _Ø_] _I_ [[ESCAPE] _Ø_] _I_

The sentence contains two intonational phrases, separating the topic of the sentence from the comment. The first intonational phrase consists of two lower-level phonological phrases (see Nespor and Sandler, 1999). The phrases are signaled by the timing of the hands, and the facial intonation aligns itself with these prosodic constituents, as is the case with intonation patterns in spoken language.

In Figure 8, we see several of the characteristics described above, listed in Example (2).

(2) Composition of the complex sentence in Figure 8
  (a) The sign for SMALL represents dimensions and is thus two-handed;
  (b) Compositional facial expression: squint indicating shared information occurring on the entire topic (‘The little dog that I found last week’); and brow raise is added to squint at the end of the intonational phrase, signaling continuation/dependency
  (c) The head moves forward and down by the end of the topic and changes position for the comment, also marking a dependent relation between the two clauses
  (d) The topic and comment are contrasted by body tilt, which is simultaneously aligned with facial expression and head position, changing at the intonational phrase boundary.
  (e) The non-dominant hand, originating in the two-handed sign, SMALL, remains in the signing space to mark continuity of the topic (‘little dog’), leaving the signing space before the comment, (‘ran away’). As soon as the topic changes, the non-dominant hand configuration and location no longer signal the discourse topic (regardless of whether or not the following sign is two-handed, as it happens to be in Figure 8).

**FIGURE 9:**
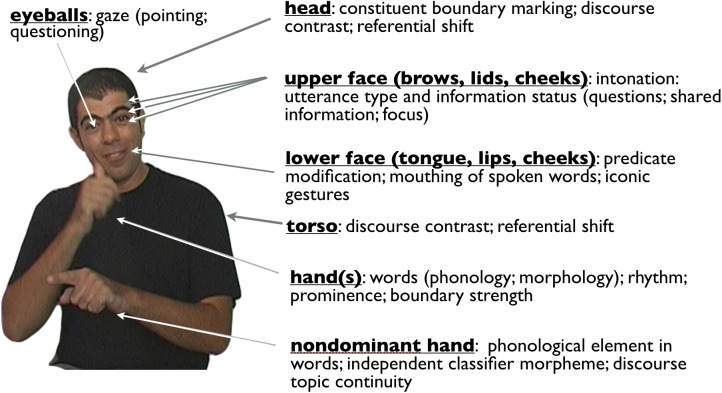
Some of the linguistic properties embodied by actions of particular parts of the body across sign languages [adapted from Sandler (2012a)].

Culling the investigations of many sign language researchers in different sign languages over the past several decades, a Grammar of the Body in sign languages is shown in Figure 9 below.

## The Composition of Language Emergence

If the structure outlined above is common to sign languages generally, then we ought to be able to witness its emergence in a very young sign language. Unlike spoken languages, which are all 1000s of years old or descended from old languages, sign languages can arise at any time, and sometimes can be caught by linguists in the act of being born. Al-Sayyid Bedouin Sign Language (ABSL), a language my colleagues and I have been investigating, began with four deaf siblings and their family, about 90 years ago (Sandler et al., 2005, 2014). The village, today numbering about 4,000, of whom about 150 are deaf, offers the exciting possibility of uncovering the fundamental ingredients of a language and tracking their development as the language is being formed.

### Al-Sayyid Bedouin Sign Language

The naïve but reasonable expectation is that new sign languages would recruit the body in a pantomimic way, so that each part of the body represents itself, ‘acting out’ events. A less naïve, but, as it turns out, equally wrong expectation – one that our team (Mark Aronoff, Irit Meir, Carol Padden, and myself) tacitly assumed at the outset – is this: In a community in which children have adult models and many hearing people also sign, complex linguistic structuring of the kind observed in established sign languages should arise very rapidly. Universal grammar principles and parameters ought to be hovering and beckoning, we expected, realized by children at the earliest opportunity.

We did not find this and, in particular, we did not find many linguistic structures that are widespread across established sign languages, such as a crystallized phonological system (Sandler et al., 2011), verb agreement (Padden et al., 2010), or a common type of complex classifier construction system. Instead, we found that the language began with a very simple base, but one which, crucially, bears the seeds of linguistic form, budding and blooming gradually and sporadically.

Armed with knowledge about language and sign language that our team brought with us, we were able to identify kernels of linguistic organization in syntax (Sandler et al., 2005), phonology (Sandler et al., 2011), and morphology (Padden et al., 2010; Meir et al., 2010), on their way to becoming more conventionalized and complex (see Aronoff et al., 2008; Sandler et al., 2014 for overviews). On the whole, we found that language begins with a good deal of variation, converging on conventionalized form gradually, and at different rates for different properties (Meir and Sandler, in press).

In the analysis of two young sign languages of equal age, ABSL and ISL, I follow the outside-in paradigm that works from the body to linguistic organization, and not the traditional paradigm, which is the other way around. ABSL, a village sign language was first conceived and developed with little outside influence, unlike ISL and similar deaf community languages.^17^ The first generation of ABSL deaf people (four siblings) and the older members of the second generation had very little or no exposure to any other language, spoken or signed. Younger signers of the second and later generations had exposure to ISL, but the amount and quality of exposure, and of influence on their language, varies greatly, depending on the educational, family, and social environment of each individual.^18^ In ABSL, each age group recruits more of the body for different linguistic functions, adding complexity concomitantly in the organization of body and language.

In a videotape of a story told by one of the first four signers, already in his 60s at the time (and deceased before we began our research), the entire body is active at the outset, but not in a linguistically organized way. Only the hands are recruited linguistically, symbolizing concepts as signs. The first unit to emerge in language, then, is the word. The whole body is involved in enacting events pantomimically, so that we have a contrast in the story between HIT, a manual sign that still exists in ABSL, and ‘strike,’ a whole-body enactment of striking someone with a sword. With a few exceptions, each proposition in the narrative, separated by pauses, consists of a single sign representing a person, object, or action, or two-sign combinations representing a verbal expression and an argument.

In a carefully coded study of narratives of two older second-generation signers and two younger second generation signers (Sandler et al., 2011), we found that older second generation signers produce longer strings than those of the first generation man, including coordinated events as well as a rough topic-comment structure, in which constituents are separated by pause and movement of the head. Younger second generation signers add systematic, linguistic facial expressions which indicate the type of relation holding between constituents, much as the brow raise indicates dependency in the ISL example (Figure 8). In other words, recruitment of the head and then the face for non-pantomimic/affective purposes adds increasing complexity to linguistic organization as well.

**FIGURE 10:**
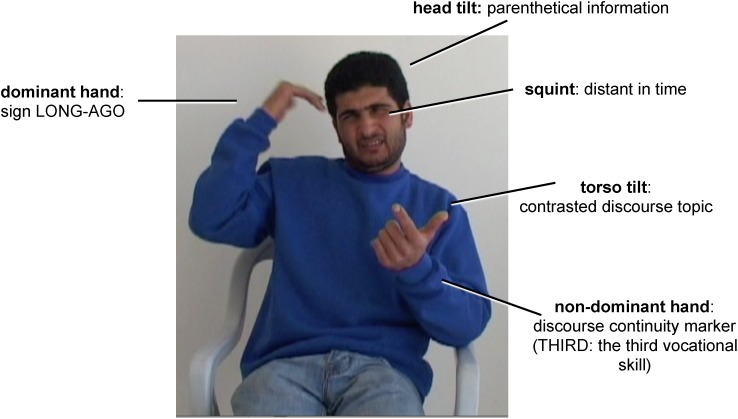
Age Group IV ABSL signer, simultaneously conveying words and discourse relations by different bodily articulations.

The narrative of a third generation signer (Age Group IV in Tables 1, 2) is more complex still, in both body and linguistic organization. He is the son of a deaf mother and is the oldest of 5 deaf siblings. He has had considerable exposure to ISL, but can distinguish ISL from ABSL in his own signing, and we found only one ISL sign in his 12-min narrative. We consider him bilingual). The signer tells of his enrollment in a vocational school, and of choosing a vocation to study there. The body is divided in much the same way as in ISL Figures 8, 9. The stretch he is signing means, “The third vocation [to choose from at the vocational school] was welding. Long ago, my father was a welder….”. The still shot in Figure 10 below was extracted from the parenthetical expression beginning with ‘Long ago.’ Each hand performs a different role; the head, torso, and face are independently recruited to provide relevant linguistic information in a simultaneous bodily configuration that is typical of sign languages (details in Sandler, 2012a).

Table 1 shows the overall picture of the emergence of linguistic structure in ABSL [where roman numerals refer to age groups, from oldest (I) to youngest (IV)].^19^ In this small but carefully documented study, there is a direct correlation between this increasing linguistic complexity and complexity in the use of the body for these functions, seen in Table 2. The hands are first – showing the human propensity for symbolization. Adding the head indicates constituency larger than the word, especially those that are connected to a following constituent, as in lists and coordinate structures. When the face is added systematically in Age Group III, illocutionary force (e.g., questions vs. declaratives) and embedding/complex sentences make their appearance. In Age Group III, the torso marks larger constituents with wider scope in the discourse, distinguishing different perspectives and referents, and the non-dominant hand establishes the discourse-level topic and keeps it in the common ground. Adding articulators whose movements entail larger spatial volume contributes to more and more sophisticated structuring of a whole discourse.

**Table 1:** Linguistic complexity in each age group [adapted from Sandler (2012a)].

Age group (Older to Younger)	Words	Complex sentences	Discourse reference/Information structure
I	Signs		
II	Signs	Unsystematic clause linking	
III	Signs	Complex sentences – embedding	Illocutionary force Parentheticals Referential shift
IV	Signs	Complex sentences - two degrees of embedding	Illocutionary force Parentheticals Double referential shift (contrasting referents) Topic continuity

**Table 2:** Recruitment of bodily articulators for linguistic functions across age groups in ABSL [adapted from Sandler (2012a)].

Age group (Older to Younger)	Hands	Head	Face	Torso	Non-dominant hand
I	X				
II	X	X			
III	X	X	X		
IV	X	X	X	X	X

We can only find the emergence of linguistic forms in new sign languages,^20^ and we can track them most clearly by observing the recruitment of parts of the body. Were we to restrict ourselves to a model of language as computation in the mind, in which ‘externalization’ by the body is of secondary importance, we would miss these generalizations entirely.

### Support From Israeli Sign Language (ISL), Another Young Sign Language

The ABSL studies rely on a small number of participants, because adult native signers of the language are so preciously few, and the results must be taken as preliminary. Israeli Sign Language is much less limited, both in the size of the deaf population (estimated at about 10,000)^21^ and in signers’ availability and flexibility. At the same time, this language arose under very different conditions, and can be considered a Creole of many substrates but no superstrate (Meir and Sandler, 2008).^22^ Studies, some of them ongoing, show consistent and quantifiable correlations between the increasing organization and integration of bodily articulations and of linguistic structure in this language (Stamp and Sandler, 2016; Dachkovsky, 2017; Dachkovsky et al., accepted).

For example, Dachkovsky (2017) studied the emergence of relative clause marking across three age groups in ISL. In this language, relative clauses are marked with non-manual signals: eye squint and forward head movement. Dachkovsky found that the oldest age group often recruited only one of the markers (typically, head position) and aligned it with the noun of the relative clause alone. In a task eliciting a response corresponding to, ‘The girl who is riding a rocking horse is eating ice cream,’ older signers who produced head movement tended to align it only with the noun, ‘girl.’ The younger age group reliably recruited both markers (squint and forward head movement), and aligned them with the whole relative clause –‘the girl who is riding a rocking horse’ – to form a constituent. The third age group performed like the second, except that the intensity of the signal was reduced, as is often the case in grammaticalization.

Another study (Dachkovsky et al., accepted) is based on spontaneous narratives, and investigates the bodily marking of discourse structures in 2 min of narrative in three age groups of ISL signers. The data were analyzed according to different degrees of discourse complexity, according to a relational hierarchy successfully used for measuring complexity and its acquisition in spoken languages. The hierarchy entails increasingly complex relations among constituents, both within and across propositions.

By comparing the bodily coding with discourse relations expressed, the study found that younger signers convey significantly more complex relations than older signers, and that the organization of the body to mark relations becomes more systematic. For a given relation, older signers are more likely to use different bodily markers, alone or configured together with another marker – specifically, tilt or shift of head or torso, alone or in combination – with no consistency. For the same relation, younger signers show a striking tendency to converge on a single articulator (either the head or the torso) and position, potentially freeing up other articulators to mark a different relation simultaneously. Reduction effects are also discernible in younger signers, and their distribution is still being analyzed.

By studying language emergence with the body as evidence, we have been able to arrive at generalizations regarding increases both in systematicity and in explicit marking of distinctions among different linguistic functions and relations, as a language develops over time. The generalizations described here, regarding higher levels of structure, are the only empirical evidence of language emergence available, and the Grammar of the Body provides a point of entry into the process.

### What Is Compositional About It?

What is compositional about this Grammar of the Body? The compositionality principle is restated for convenience:

(1′) The compositionality principle (Szabó, 2012, p. 71).
  The meaning of a complex expression is determined by the meanings its constituents have individually and the way those constituents are combined.

**FIGURE 11:**
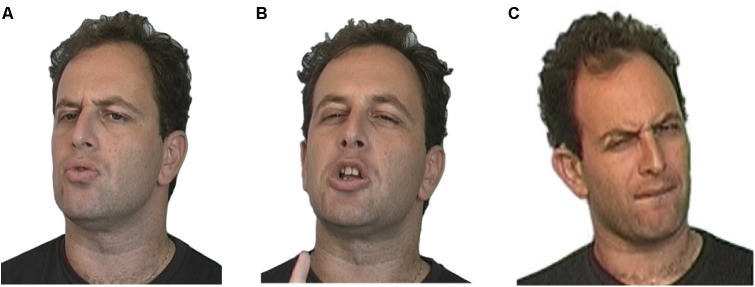
Recombining components of facial intonation in Israeli Sign Language. **(A)** Furrowed brow for wh-question. **(B)** Squint for shared information. **(C)** Furrowed brow and squint for a wh-question about shared information (following Sandler, 1999b).

What we have not yet demonstrated clearly is that the components are recombinable, adding predictable meaning in each recombination (see Talmy, 2003 on recombinance). A clear example of recombination is found in the components of intonational facial expression. Figure 5 showed that combining the raised brows of a yes–no question with the squint of shared information renders a simultaneous manifestation of the two. Similarly, adding shared information to the lowered brows intonation of a wh- (content) question, renders a simultaneous combination of those two bodily expressions, as shown in Figure 11.

In the same way, the non-dominant hand can represent any whole sign, classifier morpheme, or numeral, and its domain is determined by the topic held in the common ground within a stretch of discourse. Torso tilts can contrast information of different kinds, from foreground/parenthethical information to different discourse referents, and the information structure determines its distribution. In these ways, the components of the body combine and recombine to convey complex information in sign languages.

It has long been observed that signers of different, unrelated sign languages can strike up a conversation and understand one another (e.g., Supalla and Webb, 1995; Newport and Supalla, 2000; Zeshan, 2015). Here we see that the use of the body, intricately orchestrated in similar ways across established sign languages, together with similar strategies for iconic symbolization, provide an envelope for understanding.

**FIGURE 12:**
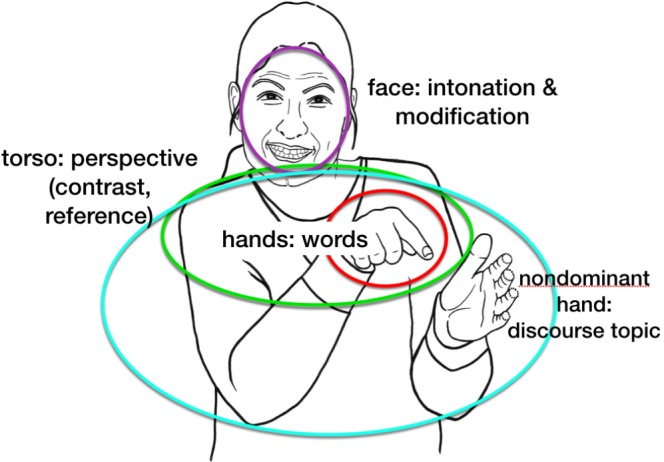
Hierarchical organization of body and discourse components.

The overall picture that emerges suggests a hierarchy, in which smaller units of language are conveyed by smaller articulators and larger ones are signaled by larger (or wider reaching) articulators, schematized in Figure 12.

The schema is intentionally broad, in order to capture generalizations that are themselves broad. As noted, the torso conveys contrasts both between referent perspectives and between topics under discussion, and torso movement can be accompanied by or reduced to head or eye movement. The non-dominant hand, in addition to signaling topic continuity, can remain in the signing space for purely prosodic reasons, not related to meaning (Nespor and Sandler, 1999). Nevertheless, this schema captures fundamental and testable relations between language and its bodily manifestation in sign languages. Where, then, did this system come from?

## Gesture

The flourishing field of gesture studies converges with this line of inquiry, investigating the properties both of gestures that accompany speech and of silent gesturing by hearing people in experimental tasks (e.g., Efron, 1941; McNeill, 1992; Goldin-Meadow, 2003; Kendon, 2004; Müller et al., 2013, 2014; Seyfeddinipur and Gullberg, 2014; Church et al., 2017; Goldin-Meadow and Brentari, 2017)^23^. We now can state unequivocally that everyone in every culture gestures. The literature is replete with examples in which gesture adds imagistic and informative, content to the message that is non-redundant – not present in the speech signal (e.g., McNeill, 1992 and many others). Most of the signal is extra-linguistic in the strict sense that it typically does not explicitly interact with the grammar, although it can be influenced by specific languages (Kita and Özyürek, 2003; Gullberg, 2011). That such elaboration is part of our universal language faculty is confirmed by the fact that signers, whose hands are occupied in the transmission of words, simultaneously produce iconic gestures with their mouths (Sandler, 2009).

Gestures can also interact more directly with linguistic structure in some cases. For example, pointing gestures are intimately integrated into speech, so that the referent of a deictic expression such as *that chair*, is unclear without pointing (Fricke, 2014).^24^ Similarly, speakers, like signers, can set up topics in space and refer back to them with gesture, or use body position for reference. In a treatment of speech act control, Landau (2016) suggests that reference can be specified solely by the position of the body. A scenario he proposes is shown in (3). In the example, the body position (body shift, when the speaker turns to the girls) identifies the intended addressees.

(3) Body shift to evaluate addressee (Landau, 2016)
  Dad and mom are reading in the living room. Jen, the older daughter, is there too. The little boys and the little girls are in the kids’ room, making a hell of a lot of noise. Dad tells Jen to go tell the *boys* to be quiet. Mom tells Jen to go tell the *girls* to be quiet (they are not aware of each other’s orders). Jen walks over to the kids’ room and says: **“[To the boys:] Dad said to be quiet, [turning to the girls] and mom did too.”**

Landau’s semantic analysis attributes the addressee function to linguistic structure, but assumes that evaluation of the addressees (the boys and the girls, respectively) belongs to extralinguistic pragmatic context. However, his analysis overlooks the fact that the body gesture is a visible signal and as such it is part of the utterance (see Kendon, 2004). In that sense, it differs from ambient pragmatic knowledge that is not signaled. Therefore, there is another possible interpretation: that bodily gesture is part of the linguistic expression. This shift in body position is reminiscent of role shift in sign languages (see the section on torso, above).

Most gesture studies attend exclusively to the hands. One exception is Birdwhistell (1970), who suggests that actions of the head and torso indicate person (first or second), prosodic boundaries, and other functions (see Kidwell, 2013 for an overview). Another is Calbris’ detailed study of Parisian gestures (Calbris, 1990), which shows that the face and the hands can separately contribute information to a configuration containing both. This is the essence of compositionality.

Adam Kendon, the father of contemporary gesture studies, in early work, clearly proposes that the physical domain of gesture is the entire body. Kendon wrote:

“Just as the flow of speech may be regarded as a hierarchically ordered set of units, so we may see the patterns of body motion that are associated with it as organized in a similar fashion, as if each unit of speech has its “equivalent” in body motion… Each speech-unit is distinguished by a pattern of movement and of body-part involvement in a movement. The larger the speech unit, the greater the difference in the form of movement and the body parts involved” (Kendon, 1972, pp. 204–205).

It is not hard to see a relation between use of the body in co-speech gesture and in sign languages as schematized in Figure 12. Yet clearly, the two systems are not the same. Gesture is typically optional with speech, and actions of the body rarely comprise explicitly linguistic constructions themselves, nor are they nearly as systematic and complex as they are in sign languages (McNeill, 1992; Özyürek, 2017). Moreover, gestures that accompany speech can only be fully understood with speech, which results in a complex – and, most likely, compositional – interaction between speech units and body units.

A detailed comparison between sign language and gesture would take us too far afield (but see, e.g., Janzen and Shaffer, 2002; Müller, 2018). However, the rich gestural scaffolding that Adam Kendon describes apparently taps into the same Grammar of the Body that underpins sign languages (see Kendon, 2012). Sign languages develop the components into fully fledged, rule-governed, compositional linguistic systems. Goldin-Meadow and Brentari (2017) conclude that language (in either modality) incorporates gesture, and that the two must be studied together.

## Roots of Compositional Expression in Intense Emotional Displays

All established contemporary human languages, spoken or signed, have a remarkable, creative, and productive range of expression, thanks in no small part to compositionality. Is this compositional structure in human language, so faithfully manifested in the body, alone in nature? Do other species possess it? Is it part of the language faculty alone, or might it have roots in other communicative systems of our species? The next section discusses some current issues in language evolution as context. The section following that presents evidence that human expression of intense emotion has compositional characteristics, suggesting a propensity for compositional expression in humans that is far more ancient than language.

### Evolution of Language: Some Key Ideas

The field of language evolution has grown to encompass a vast body of research over the past several decades. I make no attempt to do it justice here, instead offering below only a few broad comments as context.

One widely held view is that the mental computational ability of humans to produce discrete infinity, or open-endedness, in language results from recursive application of Merge, an operation that combines two syntactic units to form a new syntactic unit. Proponents of this view hold that this single property distinguishes human language (the faculty of language in the narrow sense – FLN) from communication systems of other animals (Hauser et al., 2002).

According to one view, the computational ability attributed to FLN has no evolutionary precursor and is due to a small mutation resulting in rewiring of the human brain (Chomsky, 2007). It follows that the only reasonable direction for linguistic investigation to take is to develop the best theory to characterize this ability in contemporary humans. A different paradigm accepts the centrality of FLN in language evolution, but proposes that the evolution of this mental computational ability can be traced from cognitive (not communicative) systems of other species. Seyfarth and Cheney (2014) argue that, while there is only scant evidence for hierarchical or recursive structure in communication systems of other species, there is elaborate hierarchical structure in social cognition, particularly of non-human primates, and it is this cognitive underpinning that could have provided the basis for language. In a cogent review of a recent book by Berwick and Chomsky (2016), Fitch argues that “animal cognition offers richer parallels and potential precursors to human thought and concepts than does animal communication” (Fitch, 2017, p. 603). He reasons that if recursive computation is a cognitive capability, then it makes sense to seek its evolutionary roots in the cognitive abilities of other species.

The uniqueness of recursion as the sole property responsible for open-endedness (‘discrete infinity’) has recently been questioned by Meir (2018). She demonstrates that a different kind of open-endedness – topic open-endedness – is a defining characteristic of human language, though it is not facilitated by recursion. Topic open-endedness refers to our uniquely human ability to express an endless variety of situations, thoughts, and ideas, real or hypothetical. She argues that, while Al Sayyid Bedouin Sign Language, an emerging language, does not have syntactically marked recursion at the outset (Sandler et al., 2011), it does have all the critical properties responsible for topic open-endedness – properties that are not present in communication systems of other species – symbolization, meaning extension, predication, negation, and compositionality.

Compositionality, the property that is the focus here, is present in all languages, including very young ones like the earliest forms of ABSL (see Sandler, 2012a for sample utterances of a first generation signer). Compositionality emerges in real time in iterated learning laboratory experiments with visually perceived stimuli, in which participants tend to extract recombinable components from holistic symbol transmission, and to assign meaning to them, from “generation” to “generation” (see Smith and Kirby, 2012 for an overview).

We find robust compositionality in the bodily division of labor in sign languages and in gesture, as shown in earlier sections. In the next section, we extend the body-as-evidence approach to address the evolution of this property. Our approach contrasts conceptually with the view that the “externalization” of language by the body is of secondary importance in language evolution (Berwick and Chomsky, 2016). The body-as-evidence view is compatible with Fitch’s (2017) position that externalization is important in understanding language evolution, but for different reasons. Fitch argues that externalization by the body is important because it can provide critical clues to computation and processing required by language. Here we see the body as manifesting, and thus revealing, compositional properties of language, directly. The experiment described below explores the human propensity for compositionality in a kind of bodily expression that is far more ancient than language: intense emotion.^25^

### Corporeal Emotional Displays of Athletes and Their Interpretation

Certain emotional configurations of facial expression (Ekman, 1992) and of body posture (de Gelder et al., 2015) are reliably interpreted in the same way. This shows that they are communicative (e.g., Fridlund, 2014). But does this form of communicative expression consist of holistic gestalts of face and body displays? Or is it compositional – like language?

Some researchers hold that facial configurations in particular are holistic or non-compositional, e.g., that all the facial actions contributing to an angry face or a happy face form a conglomerate (Ekman, 1992). Others suggest that each particular action of different parts of the face contributes its own meaning, in a structure that is compositional in nature (Russell, 1997; Scherer and Ellgring, 2007). Aviezer et al. (2012, 2015) are among the few who have considered both the face and the body together. In a study of emotional displays of athletes, they found the body to be a more reliable indicator of valence (positive or negative emotion) than the face, and they concluded that the face is ambiguous (Aviezer et al., 2015).

In our own recent work, we ask a different question. Within displays of intense emotion, we ask: Is it possible to identify emotions or emotional states associated with individual face/body features that contribute to the interpretation of the overall display? To probe this question, we investigated the displays of intense emotion by athletes who have just won or lost a competition (Cavicchio and Sandler, 2015; Cavicchio et al., 2018).

We select such displays first because they are intense and complex, reacting to the result of a high-stakes competition in which athletes have invested a huge amount of their lives. The intensity of the displays makes coding more straightforward, and their complexity provides a rich array of features for analysis. Second, by selecting the moment at which the athletes realize that they have won or lost, we are able to study displays which are more likely to be spontaneous and genuine, and not filtered by convention.

We began by minutely coding facial and bodily features of displays in over 300 photographs, using the Facial Action Coding System (FACS, Ekman and Friesen, 1978) for face, and a body coding system that we created. In our first study (Cavicchio and Sandler, 2015), we identified the features which statistically cluster together in victory displays and in defeat displays, respectively, revealing displays prototypical of each. Our second study (Cavicchio et al., 2018) presented participants with a total of 184 photographs of athletes: 49 displaying prototypical victory displays, 58 with prototypical defeat displays, 36 with ‘mixed’ displays, and 41 photos of athletes in non-competitive contexts, displaying neutral face and body.

We asked 84 participants to identify emotions or emotional states and their intensities in each display. Specifically, they were asked on a sliding scale of 0 to 100: “To what extent does the person in the image feel submissive/ashamed/sad/disappointed/frustrated/angry/happy/proud/dominant?”

**FIGURE 13:**
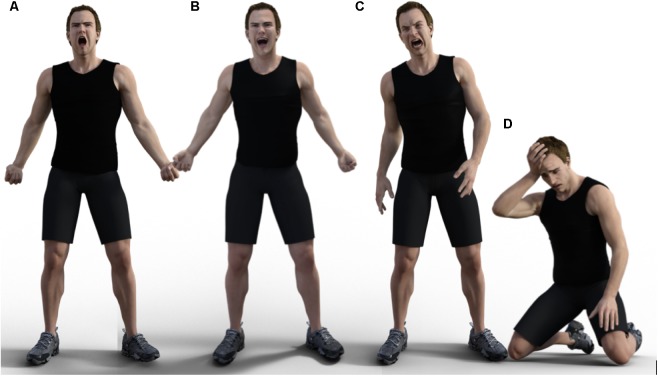
**(A)** dominant: [standing, upper body back, stretched mouth, contracted upper face]. **(B)** Dominant plus happy, same as **(A)** plus [lip corners up] and [shoulders back]. **(C)** Dominant plus angry, same as **(A)** plus [lip corners down] and [asymmetric upper body]. **(D)** submissive: [kneeling, upper body forward, hand/s to head or face, mouth slightly open, lip corners down, inner brows raised]. Figures created by Daniel Landau.

We found that the most salient categories were dominance and submission, each associated with its own block of face and body features which complemented each other in the two major categories. Dominance judgments correlated with upright posture, contracted upper face, mouth open and stretched, and clenched fists (see Figure 13). Submission correlated with prostrate posture (kneeling or lying down), head down, face covered by the hands or otherwise not visible.

Within these broad conglomerates, positive or negative emotions could be identified by looking at individual features or feature groupings. For example, [lip corners up] (smiling mouth) was deemed happy or proud and [lip corners down] was associated with the negative emotions: sadness, disappointment, frustration, and anger. We found that [lip corners down] distinguished those negative emotions from other negative emotions which express resignation and did not have this feature: submissiveness and shame.

Individual features related to the position of the upper body also contribute to interpretation. The feature [forward upper body] was associated with the emotions submission, shame, and sadness. These emotions are grouped by Ortony et al. (1990) as evaluative disapproval and focusing on self, which we interpret as resignation. The feature [asymmetrical upper body] was significantly associated with emotions related to disappointment, frustration, and anger, grouped by the same authors as reactions to goal obstruction. The position of the upper body, then, distinguishes resignation from resistance to goal obstruction.

Our results were tallied statistically from complex displays, and included only emotions rated by participants as strongly expressing a given emotion on the emotion scale.^26^. While the pictures of athletes were complex and did not necessarily reflect all typical constellations together, we can infer that the strongest dominant postures were typically characterized by all highly rated features together, and this is confirmed by the earlier analysis of these pictures in terms of features that clustered with victory (a typically dominant display) and similarly with defeat (a typically submissive display). Based on the findings in Cavicchio et al. (2018), we have now created computer-generated 3D images that reflect abstract representations of emotional states consisting of all the features that were significantly associated with them, and that pinpoint distinctions and refinements made by individual features or feature groupings on this basis.

Figure 13 below shows images of displays that are (A) dominant, (B) dominant and happy, (C) dominant and angry, and (D) submissive and resigned. The main features associated with each one, and distinguishing them from each other and other displays as elaborated in Cavicchio et al. (2018), are listed in the figure caption. We can think of these images, derived from participant ratings, as composite realizations of typical mental representations of these emotional states.

Compositionality of emotional displays reveals ancient underpinnings of compositional communication that are potentially relevant to the evolution of language. However, the use of the body in emotional displays does not correspond in any direct way to its use in language. Our results do not suggest that the Grammar of the Body sketched in relation to sign languages corresponds to the use of the body in the expression of emotion, nor would we expect it to. Language is not emotion. What the two have in common is communicativeness and complex compositionality not found to date in other species.

Interpretation of emotional displays is highly context dependent. In an experiment in which actors performed contextualized narratives with nonce speech, Dael et al. (2012) found that body forward signals hot anger, while in our studies of sports competitions, torso forward is associated with resignation and submission. The difference might be attributable to differences in coding categories (whether or not ‘forward’ entails bending at the waist), or to different interpretations of the same feature in different contexts. The answer awaits future research. The interpretation of linguistic expressions is also somewhat dependent on context, but conceivably to a much lesser extent.

The complex emotional expressions described above bear the human trait of compositionality, and differ strikingly from communicative expressions of other species, as far as we know (see footnote 4). We do not yet know whether there are constraints on the combinations of face and body actions, nor do we know how productively the components that comprise them can be manipulated and recombined to form new messages. Such possibilities and comparisons of different kinds of compositional communication offer a new spectrum of research possibilities.

## Summary and Conclusion

The relation between mind and body has been debated by philosophers for centuries (Robinson, 2017), because the issue is central to understanding human nature. Scientific investigations of spoken and signed language include the description of bodily articulation (particularly in phonology in spoken language, and of the whole body in sign language). But since the language faculty is often seen as a property of mind alone, the role of the body is viewed as secondary for understanding the essential principles governing language. Here we propose a change, by showing that the body does provide evidence for key properties of language and its emergence.

If successful, the approach proposed here will encourage several directions of research, some of them already underway. A nuanced theory of the Grammar of the Body will make informed predictions, which can be empirically tested, about structures that are likely to occur in all established sign languages, and will uncover differences as well. Such structures can also reflect the underlying composition of spoken constituents, as we have seen in connection with sign language and gesture – from the semantic components of words to reference, complex propositions, and higher levels of discourse. Detailed comparisons between sign languages and their gestural roots, to some extent shared by all, can ensue, following Kendon’s insights (see Gesture).

Sign languages provide contemporary, empirical evidence for language emergence, in populations of contemporary humans. These emerging sign languages are the only empirical source of evidence for identifying the bare essentials of language that emerge without any model and for the development and conventionalization of complex structures across generations. The Grammar of the Body model, and more refined measures of body and language efficiency and complexity sketched in section Support From Israeli Sign Language (ISL), Another Young Sign Language, can be developed and elaborated to explore the emergence of other sign languages and their development over time.

There is no doubt that visible bodily actions evolved as part of our communicative endowment, and evolutionary biologists take the body seriously in understanding language evolution (e.g., Donald, 1993; Fitch, 2010, 2017). We are now developing a test of our preliminary findings about the compositionality of bodily displays of emotion by experimentally manipulating the components and investigating the resulting interpretations. The role of context in organizing and interpreting emotion displays vs. linguistic expressions also offers fertile ground for future comparison and characterization of these systems.

Taken together, evidence from spoken language, sign language, language emergence, co-speech gesture, and the communicative expression of emotion demonstrates that compositional communication in all domains is an inherent human trait. We have been able to arrive at this conclusion by admitting the body as evidence for the nature of language.

## Ethics Statement

The research was fully approved by the European Research Council’s Ethics Committee.

## Author Contributions

The author confirms being the sole contributor of this work and has approved it for publication.

## Conflict of Interest Statement

The author declares that the research was conducted in the absence of any commercial or financial relationships that could be construed as a potential conflict of interest. The reviewer BC and handling Editor declared their shared affiliation.

## Footnotes

1 A reviewer pointed out that language can be communicated without the visual component – for example, in telephone conversations. However, people use manual and facial gestures, sometimes prolifically, in phone conversations as well, and both the degree to which telephone speakers produce added linguistic information as compensation, and the degree to which information is lost to the perceiver in these situations, have yet to be studied rigorously. Direct deictic expressions such as ‘that’ and ‘there’ must be accompanied by gesture, and are infelicitous in telephone conversations. That bodily gesture is a basic component of linguistic communication is attested by the fact that congenitally blind people also gesture when they speak (Iverson and Goldin-Meadow, 1998).

2 Throughout, reference to humans refers only to contemporary homo sapiens and not to any predecessors.

3 Exceptions to the tendency to ignore the body are the disciplines of phonology and intonation, which commonly attribute universal generalizations to the nature of the articulatory and perceptual systems and their transmission and acquisition (e.g., Browman and Goldstein, 1992; Archangeli and Pulleyblank, 1996; Blevins, 2004, 2012; Gussenhoven, 2004).

4 Other species, notably, birds, have combinatorial structure in their communication – elements combine and recombine – but the components and their recombinations are usually not considered meaningful (e.g., Wohlgemuth et al., 2010). There is some literature demonstrating limited compositionality based on laboratory experiments manipulating tones in birdsong (Suzuki et al., 2017). Non-human primates communicate multi-modally (Liebal et al., 2013), but the authors do not present evidence of compositionality. Arnold and Zuberbühler (2012) write that two-part meaningful vocal components recombine in non-human primates, and a single vocal signal in putty-nosed monkeys appears to modify meaning in combination with other components (Schlenker et al., 2016). To my knowledge, no evidence has been presented of complex combinations with reliable interpretations in non-human species.

5 I thank a reviewer for noticing this.

6 I thank a reviewer for bringing this article to my attention.

7 Frishberg (1975) shows that signs can become more arbitrary and less iconic over time, and Aronoff et al. (2005) show that iconic types of morphological complexity are common across sign languages, while arbitrary derivational morphology (often the result of grammaticalization in spoken languages) is much more rare in these young languages.

8 In this discussion, we do not deal with affective or emotional facial expressions.

9 There has been some debate as to whether these facial signals are components of syntactic or intonational structure. My colleagues and I have argued at length that they align rhythmically with prosodic constituents, are not isomorphic with syntactic constituents, and, like spoken intonation, perform the pragmatic role of organizing information structure (e.g., Nespor and Sandler, 1999; Dachkovsky et al., 2013; Sandler, 2010; Sandler et al., accepted). As such, although they interact with syntax, like prosodic signals in any language, they are fundamentally distinct from syntax.

10 The mouth has many additional roles in sign languages, such as creating iconic gestures, much as the hands do when accompanying speech (Sandler, 2009). See also Boyes-Braem and Sutton-Spence (2001) on functions of the mouth in different sign languages.

11 Wilbur and Patschke (1998) deal with a third kind of torso displacement in ASL: leans on the front/back axis. They show that body leans toward or away from the addressee indicate inclusion and exclusion of the addressee. The precise functions and interactions between leans, tilts, and shifts in different sign languages suggest themselves for future research.

12 Torso shifts and tilts can be reduced to movement of the head or eye gaze only. Distribution of these reduced signals is left to future research.

13 See Kita et al. (2014) for a discussion of the status of the non-dominant hand in signs.

14 The non-dominant hand can also function as a meaningless phonological element, i.e., as a meaningless element that spreads within prosodic constituents (Nespor and Sandler, 1999).

15 Space does not permit discussion of the classifier construction system here, in which the two hands can each represent a different classifier morpheme in the same expression (Supalla, 1986; Aronoff et al., 2003; Benedicto and Brentari, 2004; Sandler and Lillo-Martin, 2006; Janke and Marshall, 2017 among many others).

16 See Lillo-Martin and Klima (1990) and Cormier (2012) for a seminal treatment of referential loci and pronominal signs.

17 I adopt the distinction between village sign languages, which arise in insulated, homogeneous villages with a deaf population, and deaf community sign languages, which are conventionalized in heterogeneous populations, often in schools for deaf children (Meir et al., 2013).

18 It is very difficult to find a pristine language, utterly untouched by other languages. See Meir et al. (2013) and Meir and Sandler (in press) for descriptions of the characteristics of the two language communities, and the impact of these differences on the linguistic structure.

19 In all of our work on language emergence, we adopt Labov (1963) Apparent Time Hypothesis, more recently supported by Sankoff (2006). Since a person’s language changes little after the critical period, we can reliably identify diachronic change by synchronically documenting the language of succeeding age groups.

20 A well-known example of a young sign language is Nicaraguan Sign Language (NSL, e.g., Senghas and Coppola, 2001). Some accounts of this language claim that linguistic structure emerges very rapidly (Kegl et al., 1999), and this impression is common in references to NSL, even if most NSL researchers themselves are typically more cautious. In our work on Al-Sayyid Bedouin Sign Language, we stress that its linguistic structure emerges, but gradually. It is difficult to compare NSL with either ABSL or ISL, because the methods of data elicitation and analysis are quite different.

21 The figure of 10,000 deaf ISL signers is considered a conservative estimation, based on enrollment in educational programs for deaf and hearing impaired children, and on figures of the health and welfare ministry regarding disability stipends (p.c. Yael Kakon, Director of the Institute for the Advancement of Deaf Persons in Israel). There are no official population figures available.

22 See Meir and Sandler (in press) for a comparison of variation and conventionalization in these two languages.

23 See Müller, 2018 for a current detailed comparison of sign and gesture.

24 Speakers can point with many parts of the body, not just a hand, but in true deixis, they must use a visible gesture of some sort.

25 Some have suggested that visible bodily forms of expression – sign, gesture, or pantomime – preceded speech in evolution (Armstrong et al., 1995; Corballis, 2003; Arbib, 2012), an issue that is orthogonal to the present discussion of communicative bodily compositionality. But it should not go unnoticed in this context that in hearing as well as deaf contemporary humans, both the mouth and the hands are intricately, profusely, and simultaneously involved in communicative expression (Boyes-Braem and Sutton-Spence, 2001; Sandler, 2009).

26 In our analysis, we collapsed FACS action units which overwhelmingly occurred together, such as those that lower the brows and narrow the eye aperture, referred to here as ‘contracted upper face.’ See details in Cavicchio et al. (2018).
